# Supplementary material for: Computational screening of the effects of mutations on protein-protein off-rates and dissociation mechanisms by τRAMD
Source: Commun Biol. 2024 Sep 17;7:1159. doi: 10.1038/s42003-024-06880-5 (PMC11408511; doi:10.1038/s42003-024-06880-5)
Supplement: Supplementary file 3 — Description of Additional Supplementary File [file 42003_2024_6880_MOESM3_ESM.pdf]

Description of Additional Supplementary Files

File name: Supplementary Data 1
Description: Experimental and computed residence times, source data for Figures 1, 2 and 3

File name: Supplementary Movie 1
Description: Representative trajectory illustrating the main dissociation path for Bn-Bs WT, which is also shown in Figures 4 and 7.

File name: Supplementary Movie 2
Description: Representative trajectory illustrating the alternative dissociation path for Bn-Bs D35A, which is also shown in Figures 4 and 7.
